# Supplementary material for: An unbiased comparison of 14 epigenetic clocks in relation to 174 incident disease outcomes
Source: Nat Commun. 2025 Dec 16;16:11164. doi: 10.1038/s41467-025-66106-y (PMC12708718; doi:10.1038/s41467-025-66106-y)
Supplement: Supplementary file 1 — Description of Additional Supplementary Files [file 41467_2025_66106_MOESM1_ESM.pdf]

Description of Additional Supplementary Files

Supplementary Data 1. Descriptive statistics for the 174 disease outcomes

Supplementary Data 2. Cox regression results for 14 clocks and 174 incident disease outcomes. Models consider either basic or full covariate adjustment in addition to stratification and interactions by sex and smoking (two-sided tests).

HR = Hazard Ratio, LCI = lower 95% confidence interval, UCI = upper 95% confidence interval, P = p-value, P_local = p-value for the Shoenfeld residual test for the predictor (clock) of interest, P_global = p-value for the Schoenfeld residual test for all predictors in the model. The case/control numbers for the smoking stratification analyses vary from those in the main models as ever/never status was based on observed pack years data (never smoked = 0 pack years), while missing pack years values were imputed prior to its inclusion as a covariate. Results are only shown for diseases where n_{cases} > 30 within strata.

Supplementary Data 3. Cox regression and logistic regression results for 14 clocks and 174 incident disease outcomes (fully-adjusted models, two-sided tests).

HR = Hazard Ratio, LCI = lower 95% confidence interval, UCI = upper 95% confidence interval, P = p-value, P_local = p-value for the Schoenfeld residual test for the predictor (clock) of interest, P_global = p-value for the Schoenfeld residual test for all predictors in the model, AUC (null model) = area under the curve for the logistic regression model with covariates only, AUC (full model) = area under the curve for the logistic regression model with covariates and the clock of interest, AUC full – AUC null = difference between the AUC (full model) and AUC (null model), P (AUC difference) = formal statistical comparison of the nested full and null AUC models.

Supplementary Data 4. Mean and standard deviation of the log hazard ratios for each clock, averaged across all 174 disease associations.

Supplementary Data 5. Linear mixed effects model output contrasting the mean log hazard ratio for each clock against GrimAge v1 (reference category). DNAmTL effect sizes were reverse coded prior to the regression analysis. All tests are two-sided,

Supplementary Data 6. Cox regression and logistic regression results for 14 clocks and all-cause mortality (fully-adjusted models, all two-sided tests).

HR = Hazard Ratio, LCI = lower 95% confidence interval, UCI = upper 95% confidence interval, P = p-value, P_local = p-value for the Schoenfeld residual test for the predictor (clock) of interest, P_global = p-value for the Schoenfeld residual test for all predictors in the model, AUC (null model) = area under the curve for the logistic regression model with covariates only, AUC (full model) = area under the curve for the logistic regression model with covariates and the clock of interest, AUC full – AUC null = difference between the AUC (full model) and AUC (null model), P (AUC difference) = formal statistical comparison of the nested full and null AUC models.

Supplementary Data 7. Subset of Table S3 showing Bonferroni significant associations where the absolute log hazard ratio is greater for the respective disease-clock association compared to the mortality-clock association.

Supplementary Data 8. Subset of Table S3 showing Bonferroni significant associations where the difference in AUC with the basic-adjusted model is greater than 0.01 (1%) and is nominally significant: P (AUC difference) < 0.05.
