# Supplementary material for: An unbiased comparison of 14 epigenetic clocks in relation to 174 incident disease outcomes
Source: Nat Commun. 2025 Dec 16;16:11164. doi: 10.1038/s41467-025-66106-y (PMC12708718; doi:10.1038/s41467-025-66106-y)
Supplement: Supplementary file 10 — Reporting Summary [file 41467_2025_66106_MOESM10_ESM.pdf]

nature portfolio

Corresponding author(s): Riccardo Marioni

Last updated by author(s): Oct 27, 2025

Reporting Summary

Nature Portfolio wishes to improve the reproducibility of the work that we publish. This form provides structure for consistency and transparency in reporting. For further information on Nature Portfolio policies, see our Editorial Policies and the Editorial Policy Checklist.

Statistics

For all statistical analyses, confirm that the following items are present in the figure legend, table legend, main text, or Methods section.

n/a	Confirmed	
☐	☒	The exact sample size (*n*) for each experimental group/condition, given as a discrete number and unit of measurement
☐	☒	A statement on whether measurements were taken from distinct samples or whether the same sample was measured repeatedly
☐	☒	The statistical test(s) used AND whether they are one- or two-sided<br>*Only common tests should be described solely by name; describe more complex techniques in the Methods section.*
☐	☒	A description of all covariates tested
☐	☒	A description of any assumptions or corrections, such as tests of normality and adjustment for multiple comparisons
☐	☒	A full description of the statistical parameters including central tendency (e.g. means) or other basic estimates (e.g. regression coefficient) AND variation (e.g. standard deviation) or associated estimates of uncertainty (e.g. confidence intervals)
☐	☒	For null hypothesis testing, the test statistic (e.g. *F*, *t*, *r*) with confidence intervals, effect sizes, degrees of freedom and *P* value noted<br>*Give P values as exact values whenever suitable.*
☒	☐	For Bayesian analysis, information on the choice of priors and Markov chain Monte Carlo settings
☒	☐	For hierarchical and complex designs, identification of the appropriate level for tests and full reporting of outcomes
☒	☐	Estimates of effect sizes (e.g. Cohen's *d*, Pearson's *r*), indicating how they were calculated

Our web collection on statistics for biologists contains articles on many of the points above.

Software and code

Policy information about availability of computer code

Data collection	NA
Data analysis	Fully annotated copies of the analysis scripts are available at: https://github.com/marioni-group/Epigenetic_Clocks_and_Disease_GS.

For manuscripts utilizing custom algorithms or software that are central to the research but not yet described in published literature, software must be made available to editors and reviewers. We strongly encourage code deposition in a community repository (e.g. GitHub). See the Nature Portfolio guidelines for submitting code & software for further information.

Data

Policy information about availability of data

All manuscripts must include a data availability statement. This statement should provide the following information, where applicable:

- Accession codes, unique identifiers, or web links for publicly available datasets
- A description of any restrictions on data availability
- For clinical datasets or third party data, please ensure that the statement adheres to our policy

The source data that were analysed during the current study are not publicly available due to them containing information that could compromise participant consent and confidentiality. Data can be obtained from the data owners. Instructions for accessing Generation Scotland data can be found here: https://www.ed.ac.uk/generation-scotland/for-researchers/access; the 'GS Access Request Form' can be downloaded from this site. Completed request forms must be sent to access@generationsco tland.org to be approved by the Generation Scotland Access Committee.

Research involving human participants, their data, or biological material

Policy information about studies with human participants or human data. See also policy information about sex, gender (identity/presentation), and sexual orientation and race, ethnicity and racism.

Reporting on sex and gender	Biological sex was defined using self-report and confirmed by genotype data
Reporting on race, ethnicity, or other socially relevant groupings	The subset of Generation Scotland with DNA methylation data is of European ancestry (based on genotype).
Population characteristics	Generation Scotland (GS) is a family-based research study comprised of 24,084 volunteers [14]. Volunteers joined the cohort between 2006 and 2011 with the majority of recruitment taking place via invitation from the individuals' general practitioners (GPs). Initially, individuals aged 35-65 years were recruited from across five geographic areas (Glasgow, Aberdeen, Dundee, Ayr and Arran). They were then encouraged to recruit family members living in Scotland to join the study. This resulted in a baseline cohort aged between 17 and 99 years. All individuals were invited to complete questionnaires and to attend a baseline clinic visit for further questionnaires and testing, resulting in a detailed collection of lifestyle, clinical, health, cognitive and sociodemographic data. The majority also agreed to provide biosamples, including blood, from which DNA has been extracted and profiled for genetic and epigenetic data. Most individuals also provided consent for researchers to access their medical records via data linkage to primary and secondary care codes.
Recruitment	See above
Ethics oversight	All components of Generation Scotland received ethical approval from the NHS Tayside Committee on Medical Research Ethics (REC Reference Number: 05/S1401/89). All participants provided broad and enduring written informed consent for biomedical research. Generation Scotland has also been granted Research Tissue Bank status by the East of Scotland Research Ethics Service (REC Reference Number: 15/0040/ES), providing generic ethical approval for a wide range of uses within medical research. This study was performed in accordance with the Helsinki declaration.

Note that full information on the approval of the study protocol must also be provided in the manuscript.

Field-specific reporting

Please select the one below that is the best fit for your research. If you are not sure, read the appropriate sections before making your selection.

[x] Life sciences [ ] Behavioural & social sciences [ ] Ecological, evolutionary & environmental sciences

For a reference copy of the document with all sections, see nature.com/documents/nr-reporting-summary-flat.pdf

Life sciences study design

All studies must disclose on these points even when the disclosure is negative.

Sample size	Sample size was determined as those who passed DNA methylation QC and had data linkage to health records (n=18,859)
Data exclusions	For each of the 174 incident diseases, we excluded those with a prevalent diagnosis at the time of blood draw for DNA methylation
Replication	Replication was not conducted. Generation Scotland is currently the world's single largest published DNA methylation dataset. Whilst we would be able to replicate findings for some diseases (e.g., Type 2 diabetes has been well characterised by several cohort studies), the extent of data linkage and frequency of disease outcomes from smaller cohorts would be insufficiently powered for widespread replication.
Randomization	NA. Common lifestyle risk factors were included as covariates in the Cox models.
Blinding	NA

Reporting for specific materials, systems and methods

We require information from authors about some types of materials, experimental systems and methods used in many studies. Here, indicate whether each material, system or method listed is relevant to your study. If you are not sure if a list item applies to your research, read the appropriate section before selecting a response.

Materials & experimental systems

n/a	Involved in the study	
☒	☐	Antibodies
☒	☐	Eukaryotic cell lines
☒	☐	Palaeontology and archaeology
☒	☐	Animals and other organisms
☐	☒	Clinical data
☒	☐	Dual use research of concern
☒	☐	Plants

Methods

n/a	Involved in the study	
☒	☐	ChIP-seq
☒	☐	Flow cytometry
☒	☐	MRI-based neuroimaging

Clinical data

Policy information about clinical studies

All manuscripts should comply with the ICMJE guidelines for publication of clinical research and a completed CONSORT checklist must be included with all submissions.

Clinical trial registration	NA
Study protocol	NA
Data collection	NA
Outcomes	NA

Plants

Seed stocks	NA
Novel plant genotypes	NA
Authentication	NA
